# Nephrotoxicity Evaluation on Cisplatin Combined with 5-HT_3_ Receptor Antagonists: A Retrospective Study

**DOI:** 10.1155/2018/1024324

**Published:** 2018-05-30

**Authors:** Wen Kou, Hongyan Qin, Shahbaz Hanif, Xinan Wu

**Affiliations:** ^1^The First Hospital of Lanzhou University, Pharmacy Department, Lanzhou, China; ^2^The First Hospital of Lanzhou University, Third Department of General Surgery, Lanzhou, China

## Abstract

**Objective:**

5-HT_3_ receptor antagonist (ondansetron) has been reported to have nephrotoxic effect when combined with cisplatin in mice; however, little evidence exists in explaining its nephrotoxic effects on patients. The aim of this present study was to investigate whether 5-HT_3_ receptor antagonist could enhance or aggravate the incidence of cisplatin-induced nephrotoxicity in patients.

**Methods:**

We retrospectively reviewed 600 tumor patients which were treated with cisplatin (*⩾*60 mg/m^2^) as a first-time chemotherapy and combined with 5-HT_3_ receptor antagonist (i.e., ondansetron, tropisetron, or ramosetron, each kind of 5-HT_3_ receptor antagonist contains 200 cases) between January 2010 and December 2015. Cisplatin dosing, the baseline creatinine clearance, and other independent risk factors such as patient's age, sex, PS score, and weight associated with nephrotoxicity were evaluated in a multivariable model.

**Results:**

The incidence of Grade *⩾* 2 serum creatinine elevation in cisplatin + ondansetron group was significantly higher than cisplatin + tropisetron group (*P* = 0.04), but no significant difference was found between cisplatin + ondansetron group and cisplatin + ramosetron group (*P* = 0.3). It was also found that cisplatin dosage and tumor type were independent risk factors in the development of nephrotoxicity.

**Conclusion:**

Higher cisplatin dosage and regular use of ondansetron combined with cisplatin are more likely to increase the incidence of nephrotoxicity; tropisetron showed the relatively mild effect on kidney function, suggesting that tropisetron is a preferable alternative in the process of cisplatin chemotherapy.

## 1. Introduction

Cisplatin is one of the most widely used platinum drugs in chemotherapy regimens for patients with lung cancer and other kinds of malignancies, such as ovarian, endometrial, bladder, head and neck, cervical, stomach, prostate cancers, Hodgkin's and non-Hodgkin's lymphomas, multiple myeloma, melanoma, and mesothelioma [1]. The most severe adverse effect caused by cisplatin is nephrotoxicity. Cisplatin kidney injury is dose-duration-frequency dependent and is reported to induce acute or chronic renal impairment in 28%–42% of patients treated with cisplatin [2–5]. Currently, the pathogenic mechanism of cisplatin-induced nephrotoxicity is still not very clear.

It is well known that cisplatin is mainly excreted into the urine during the first 24 h after administration and the concentration of cisplatin in the renal cells is much higher than that in the plasma [6–8]. Therefore, it is speculated that cisplatin may have damage on the proximal tubule cells of kidney and thus increase the serum creatinine level [9]. Some researchers also demonstrated that certain transporters, such as OCT2 (organic cation transporter) and MATE1 (multidrug and toxin extrusion protein), may play an important role in the accumulation of cisplatin in renal proximal tubules [10–12]. In addition, it is reported that cimetidine, an OCT2 inhibitor, can reduce the nephrotoxicity of cisplatin in wide-type mice and in Oct1/2 knockout mice [13, 14]. Based on above evidence, it is possible that OCT2 or MATE1 mediated cisplatin accumulation in renal proximal tubules may contribute to nephrotoxicity of cisplatin.

5-HT_3_ antagonists are widely used as antiemetic agents for patients receiving highly emetogenic cisplatin-based regimens [15]. It is found that 5-HT_3_ receptor antagonists, such as ondansetron and tropisetron, are the substrate of OCT2 and MATE1 [16, 17]. Are these 5-HT_3_ antagonists risk factors in cisplatin-induced nephrotoxicity? Recently, a research answered this question, which found that ondansetron significantly enhanced renal accumulation of cisplatin and cisplatin-induced nephrotoxicity in mice [18]. Until now, clinical research concerning the potential nephrotoxic effects of cisplatin combined with 5-HT_3_ receptor antagonists is still absent. This study aims to retrospectively compare the nephrotoxicity in patients treated with cisplatin combined with 5-HT_3_ receptor antagonists, i.e., ondansetron, tropisetron, and ramosetron. The results of this study will provide useful evidence about how to select 5-HT_3_ receptor antagonists when patients were treated with cisplatin.

## 2. Patients and Methods

### 2.1. Patients

A retrospective study was conducted in the First Hospital of Lanzhou University in July, 2016 (the data were analyzed anonymously, so we did not sign the informed consent). We examined the clinical data of patients (data between January 2010 and December 2015) who received therapies including a high dose (*⩾*60 mg/m^2^) of cisplatin in the first-line chemotherapy and combined with 5-HT_3_ receptor antagonists (i.e., ondansetron, tropisetron, or ramosetron, each kind of 5-HT_3_ receptor antagonist group contains 200 cases). Patients were included, if they had pathologically confirmed malignancies, an Eastern Cooperative Oncology Group performance status (PS) of 0 to 2, and the serum creatinine level was in the normal range before the chemotherapy. Patients were excluded from the study if they had a history of cisplatin treatment or had more than one cancer.

### 2.2. Hydration and Treatment Methods

Cisplatin (*⩾*60 mg/m^2^) was administered over 60 min on Day 1 in combination with other chemotherapeutic agents, mannitol and 2000 ml of hydration. There was no difference between the groups with respect to the volume of hydration. Antiemetic prophylaxis consisted of 5-HT_3_ receptor antagonist (ondansetron: 8 mg, tropisetron: 5 mg, or ramosetron: 0.3 mg).

### 2.3. Nephrotoxicity Evaluation

Renal function was evaluated based on the serum creatinine (SCr, *μ*mol/L) level. We use the changes in creatinine clearance (Ccr) as measure of nephrotoxicity. In this study, nephrotoxicity arising from the cisplatin-containing regimen was defined as Grade 1, 2, or more Scr elevation according to Common Terminology Criteria for Adverse Events (CTCAE), version 4.0. We evaluated the association between the incidence of Grade 2 or more Scr elevation during first-time chemotherapy and the type of 5-HT_3_ receptor antagonist. Ccr was assessed using the Cockroft-Gault formula.

### 2.4. Statistical Analysis

To identify risk factors potentially associated with the patients using different types of 5-HT_3_ receptor antagonist and the difference of the incidence of Grade 2 nephrotoxicity among the three groups, all the patients divided into cisplatin + ondansetron, cisplatin + tropisetron, and cisplatin + ramosetron groups. Factors in the analysis included age (*⩾*70 vs. <70 years), PS (2 vs. 0 or 1), sex (male vs. female), weight (*⩾*70 vs. <70 kg), cisplatin dose, baseline Ccr (mL/min), Ccr after treatment (mL/min), and tumor type, these factors showing the range and mean value (except sex, PS, and tumor type). The risk factors were evaluated in multivariable analysis with the Poisson Regression Model. The risk ratio with 95% confidence interval (CI) was calculated for the independent prognostic factors. To investigate the effect of different 5-HT_3_ receptor antagonists on cisplatin-induced nephrotoxicity, we use unpaired Student's* t*-test to test the mean change percentage from baseline in Ccr between two groups.

## 3. Results

A total of 600 patients who received chemotherapy including high-dose cisplatin were eligible for the analysis. The mean age was 56 years (range: 18–81); 375 patients were male and 225 were female; most patients had a good PS of 0-1. The most common malignancies were bronchial cancer (14.5%); the mean baseline Ccr is 99.5 mL/min (range: 45.3–205.8 mL/min) and after the first cycle of chemotherapy, the Ccr is 86.3 mL/min (range: 42.2–181.6 mL/min); the mean cisplatin dose is 76.7 mg (range: 60–120 mg) (Table 1).

Cisplatin-induced nephrotoxicity was observed in 270 of 600 enrolled patients, including 195 patients with Grade 1 nephrotoxicity and 75 patients with Grade 2 nephrotoxicity. Among cisplatin-ondansetron, cisplatin-tropisetron, and cisplatin-ramosetron groups, there are 76, 66, and 68 patients who developed Grade 1 nephrotoxicity and 28, 13, and 19 who developed Grade 2 nephrotoxicity, respectively. The incidence of Scr elevation is higher in the cisplatin-ondansetron group. As for Grade 2 nephrotoxicity, we observed that there is a trend towards higher incidence in cisplatin-ondansetron than cisplatin-tropisetron group. To assess the contribution of each risk factor to cisplatin-induced nephrotoxicity, we performed multivariable analysis; the results showed that cisplatin dosage is more related to nephrotoxicity (Table 2).

**Figure 1:**
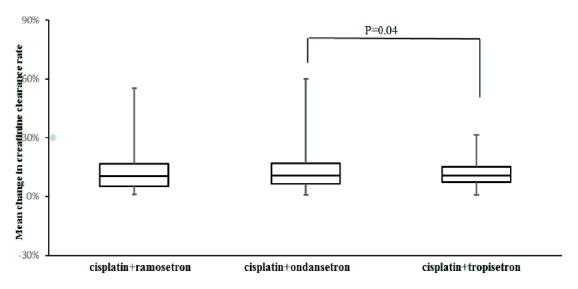
Box and whisker plot for the relations between cisplatin combined different. 5-HT_3_ receptor antagonists and the mean change in creatinine clearance rate during the first course of cisplatin chemotherapy.

To investigate the effect of 5-HT_3_ receptor antagonists on cisplatin-induced nephrotoxicity, we evaluated the mean change percentage from baseline in Ccr during the first course of cisplatin chemotherapy and observed that there is a trend towards higher incidence in Ccr change in the group receiving cisplatin and ondansetron than the other two groups. The trend strongly supports that ondansetron may aggravate the incidence of nephrotoxicity induced by cisplatin, as suggested by statistical results (Figure 1).

## 4. Discussion

In this study, we analyzed the effects of three 5-HT_3_ receptor antagonists (i.e., ondansetron, ramosetron, or tropisetron) on cisplatin-induced nephrotoxicity in patients treated with cisplatin-containing chemotherapy; our results showed that the incidence of Grade *⩾* 2 serum creatinine elevation in cisplatin + ondansetron group was the highest in the three groups. We also noticed that cisplatin dosage and tumor type were independent risk factors in cisplatin-induced nephrotoxicity. The transporter plays an important role in drug-drug interactions, which lead to the accumulation of the victim drugs in the kidney and consequently caused adverse effects [19]. Cisplatin has been characterized as a substrate for OCTs and MATEs both in vivo and in vitro, while 5-HT_3_ receptor antagonists, such as ondansetron and tropisetron, can inhibit OCTs and MATE's function. It is well known that 5-HT_3_ receptor antagonists are commonly used during cisplatin chemotherapy. Recently, a study revealed that ondansetron enhances renal accumulation of cisplatin and cisplatin-induced nephrotoxicity in mice. The studies also found that although different 5-HT_3_ receptor antagonists have similar chemical structures, nevertheless, they show notable differences in their selectivity, potency, and pharmacokinetics [20].

To our knowledge this is the first study assessing the interaction between cisplatin and 5-HT_3_ receptor antagonists in patients. In this study, we investigated 600 tumor patients which were treated with cisplatin (*⩾*60 mg/m^2^) as a first-time chemotherapy and combined with 5-HT_3_ receptor antagonists. We found that, in cisplatin + ondansetron group, the incidence of Grade *⩾* 2 serum creatinine elevation was significantly higher than that of cisplatin + tropisetron group, but this trend was not observed between cisplatin + ondansetron group and cisplatin + ramosetron group or cisplatin + ramosetron and cisplatin + tropisetron group. When comparing the mean change of Ccr before and after the chemotherapy, we observed that there is a trend that, in ramosetron group, the reduction of Ccr is higher than the other two groups, although in ramosetron group, the incidence of Grade *⩾* 2 serum creatinine elevation is lower than ondansetron group; it still has more influence on renal function than tropisetron, suggesting that tropisetron should be a preferable choice in the process of cisplatin chemotherapy.

We use multivariable analysis to assess the potential risk factors for cisplatin-induced nephrotoxicity; the results showed that cisplatin dosage is an independent risk factor in the development of nephrotoxicity; our results are consistent with another study demonstrating a higher cumulative dose increase risk for future kidney injury [21]. This finding also provide clue for us that patients treated with high-dose cisplatin chemotherapy and combined with ondansetron need to pay more attention to the incidence of nephrotoxicity.

Our study also has several limitations. First, this was a retrospective study which limited to only one department, the observation bias of the data cannot be excluded. Secondly, the patients with malignant tumor were critically of poor physical fitness; in Traditional Chinese Medicine, it is called “syndrome of deficiency of both yin and yang of kidney”; these patients' renal function was already abnormal even though their serum creatinine level was within the normal range. Thirdly, tumor patients' physiological status might influence the clearance status of cisplatin and make them more susceptible to nephrotoxicity. Multicenter controlled studies with larger samples are still needed to clarify the associations between dosing and nephrotoxicity of cisplatin and ondansetron.

## Figures and Tables

**Table 1:** Baseline characteristics of the 600 study patients.

Characteristic	All patients	Cisplatin + ondansetron	Cisplatin + tropisetron	Cisplatin + ramosetron
	(*n* = 600)	(*n* = 200)	(*n* = 200)	(*n* = 200)
Sex				
Male	375 (62.5%)	108 (54%)	114 (57%)	153 (76.5%)
Female	225 (37.5%)	92 (46%)	86 (43%)	47 (23.5%)
PS				
0-1	554 (92.3%)	185 (92.5%)	189 (94.5%)	180 (90%)
2	46 (7.7%)	15 (7.5%)	11 (5.5%)	20 (10%)
Weight (kg)				
mean	63	62	65	61
range	42–90	45–86	42–90	45–78
≥70	535 (89.2%)	174 (87%)	185 (92.5%)	176 (88.0%)
<70	65 (10.8%)	26 (13%)	15 (7.5%)	24 (12%)
Baseline Ccr (mL/min)				
mean	99.5	97.1	104.7	96.6
range	45.3–205.8	51.3–158.6	45.3–205.8	54.9–196.0
Ccr during treatment (mL/min)				
mean	86.3	92.4	93.8	72.8
range	42.2–181.6	48.9–147.4	42.2–181.6	43.5–121.1
Cisplatin dose (mg)				
mean	76.7	74	76	80
range	60–120	60–120	60–110	60–100
Age (years)				
mean	56	54	56	58
range	18–81	36–75	28–81	18–75
≥70	55 (9.2%)	16 (8.0%)	21 (10.5%)	18 (9.0%)
<70	545 (90.8%)	184 (92%)	179 (89.5%)	182 (91%)
Tumor type				
Esophageal	87 (14.5%)	10 (5.0%)	15 (7.5%)	62 (31%)
Lung	164 (27.3%)	36 (18%)	41 (20.5%)	87 (43.5%)
Gastric	31 (5.2%)	4 (2.0%)	7 (3.5%)	20 (10.0%)
Cervical	87 (14.5%)	32 (16.0%)	47 (23.5%)	8 (4.0%)
Endometrial	27 (4.5%)	20 (10.0%)	4 (2.0%)	3 (1.5%)
Bronchial	173 (28.8%)	99 (49.5%)	47 (23.5%)	27 (13.5%)
Others	31 (5.2%)	12 (6.0%)	15 (7.5%)	4 (2.0%)

**Table 2:** Risk ratio in multivariable analysis of potential predisposing factors for cisplatin-induced nephrotoxicity (*n* = 270).

Factor	Risk ratio	95% Cl	*P* value
Age (≥60 vs. ≤60)	0.131	0.036–0.326	0.202
Sex (male vs. female)	0.057	1.79–3.83	0.474
PS (2 vs. 0 or 1)	0.119	2.77–5.07	0.542
Weight	0.287	0.015–11.22	0.051
Baseline Cr	0.11	4.74–7.69	0.632
Cisplatin dose	0.057	2.40–7.46	**<0.001**
Tumor type			
Esophageal cancer	1.000		
Lung cancer	0.845	5.56–8.01	0.717
Gastric cancer	1.316	4.45–10.46	0.400
Cervical cancer	1.119	2.21–6.10	0.349
Endometrial cancer	0.838	3.38–12.24	0.238
Bronchial cancer	0.870	0.05–7.66	0.053
